# New XEN63 Gel Stent Implantation in Open-Angle Glaucoma: A Two-Year Follow-Up Pilot Study

**DOI:** 10.2147/OPTH.S423519

**Published:** 2023-08-04

**Authors:** Bogomil Voykov, Emil Nasyrov, Jonas Neubauer, Caroline J Gassel

**Affiliations:** 1 Centre for Ophthalmology, University Hospital Tuebingen, Tuebingen, Germany

**Keywords:** open-angle glaucoma, filtering surgery, minimally invasive surgery, glaucoma gel stent

## Abstract

**Purpose:**

The XEN gel stent was developed to reduce the risks of filtration surgery by standardizing the outflow of aqueous humor into the subconjunctival space. Recently, a modified version of the XEN63 gel stent was introduced. The goal of this study was to assess its efficacy and safety.

**Methods:**

This is a prospective, nonrandomized, observational, consecutive case series study at a single tertiary centre. Patients with open-angle glaucoma with above target intraocular pressure (IOP) despite maximal tolerated medication were included. The primary outcome was a change of median IOP. Secondary outcomes included a change in the number of medications, complete success, needling and complication rates. Success was defined as a lowering of IOP > 20% from baseline and IOP ≤ 14 mmHg. Complete success indicated that the target IOP was reached without medications.

**Results:**

Six patients were included. The median IOP decreased from 35.5 mmHg (25.0–40.0 mmHg) at baseline to 11.5 mmHg (4.0–15.0 mmHg, p = 0.03), and median IOP-lowering medication was reduced from 4.0 (3.0–4.0) at baseline to 0 (0–1.0, p = 0.03) after two years. Five patients (83.0%) had a complete success after two years. Two patients (33.0%) required a needling procedure. Three patients (50.0%) required an intervention due to symptomatic hypotony within the first three weeks postoperatively. Hypotony resolved completely or was asymptomatic after three months.

**Conclusion:**

Our study demonstrated a statistically significant reduction in both IOP and number of IOP-lowering medications. Complications were well manageable and had no long-term sequelae.

## Introduction

Recently, there has been increasing interest among glaucoma specialists in minimally invasive glaucoma surgery (MIGS).^1–3^ The XEN gel stent is a bleb-forming MIGS-device, which has shown promising results in a variety of glaucoma types.^4–7^ The XEN gel stent has been developed to reduce the risks of filtration surgery by standardizing the outflow of aqueous humor in the subconjunctival space. Initially, three different models with lumen diameters of 45µm, 63µm and 140µm were developed.^8^ Of those, only XEN45 has been commercially available. Recently, a modified version of the XEN63 gel stent has been introduced to the market. The main differences between the previous and the new versions are changes in the injector and the surgical technique. The size of the injector needle has decreased from 25G to 27G.^9^ Additionally, the off-label use of subconjunctival mitomycin C (MMC) has become a standard part of the procedure.^10–12^ The goal of this pilot study was to assess the efficacy and safety of the new XEN63 in the long term.

## Patients and Methods

This is a prospective, nonrandomized, observational, consecutive case series pilot study at a single tertiary centre. The study was conducted in accordance with the tenets of the Declaration of Helsinki. All patients gave informed consent prior to surgery. Ethical approval was granted by the local institutional ethics committee of the University of Tuebingen, project-number: 719/2020BO2. All patients were treated with the new XEN63 gel stent between July and August 2020. Only patients with primary open-angle glaucoma, pigmentary glaucoma or pseudoexfoliative glaucoma with above target intraocular pressure (IOP) despite maximal tolerated IOP-lowering medication were included. We used the new XEN63 gel stent (AbbVie Inc. North Chicago, Illinois, U.S.A.), which is identical to the model recently introduced into the market. It is composed of porcine gelatine crosslinked with glutaraldehyde. It is 6.0 mm long and has an outer and inner diameter of 170µm and 63µm, respectively. The injector has a 27G needle and is identical to the XEN45 gel stent injector.

### Surgical Technique

All surgeries were performed as solo procedure by a single experienced surgeon (B.V.) under topical anaesthesia as previously described.^4^ In the superonasal quadrant 25µL of mitomycin C (MMC) 0.2 mg/mL (5µg) were injected subconjunctivally 5mm from the limbus. A small clear cornea incision 1mm from the limbus was then made inferotemporally with a 20G side port knife. An additional side port incision was made 3 clock hours from the main incision. The anterior chamber was then filled with Healon ophthalmic viscoelastic device (OVD, Johnson & Johnson, NJ, USA). The XEN63-injector was introduced into the anterior chamber aiming at the superonasal quadrant under gonioscopic control. The injector needle penetrated the sclera into the subconjunctival space in the superonasal quadrant and the gel stent was slowly released. Finally, the OVD was removed from the anterior chamber.

In one patient, the procedure was modified. This patient already had a XEN45 gel stent with a fibrotic non-functioning bleb. In this case, a revision of the bleb was attempted. During the preparation of the conjunctiva, the gel stent was damaged and was removed followed by the implantation *ab interno* of a new XEN63 gel stent as described above. In this case, the conjunctiva was sutured with a 10–0 nylon suture and 10µg MMC (50µL) was injected at the site of the bleb at the end of the procedure.

### Pre- and Postoperative Management

Baseline IOP was assessed under IOP-lowering medication, which was then stopped two weeks before surgery. Oral acetazolamide twice daily was then given until the night before surgery. Unpreserved dexamethasone eye drops four times daily were started one week before surgery. No medication was given on the day of surgery. Postoperatively, antibiotic eye drops were given four times daily for a week and unpreserved steroids were tapered over 2 to 3 months. Patients were followed up on the first two postoperative days, as well as after 1, 3, 6, 12, 18 and 24 months after surgery.

During follow-up, if IOP was not adequately controlled, then a needling procedure was performed instead of starting IOP-lowering medication. Needling was performed under topical anaesthesia. A 30G needle was bent and used to inject a small subconjunctival bubble of mepivacaine next to the XEN gel stent. Fibrotic tissue around the gel stent was then disrupted with sweeping movements of the needle tip. The stent was gently moved to assure free mobility of its tip. Then, 25µL of 0.2 mg/mL MMC (5µg) were injected in the filtering bleb. After surgery, moxifloxacin eye drops were started on the first postoperative day four times daily for three days and unpreserved dexamethasone eye drops were tapered over 1–2 months.

### Statistical Analysis

The statistical analysis was performed with the JMP 16.0 statistical package (SAS Institute Inc., Cary, NC, USA). Data are expressed as number (percentage), mean (standard deviation [SD]), mean (95% confidence interval [95% CI]), or median (range) as appropriate. We used the Wilcoxon signed-rank test to evaluate changes in the number of IOP-lowering medications and mean IOP. Values for *p* of less than 0.05 were considered to reflect significant differences.

The primary outcome of the study was the median change of IOP after two years. Secondary outcomes included changes in the number of IOP-lowering medications, complete success rate, needling rate and complications rate.

Success of the procedure was defined as lowering of IOP > 20% from baseline and target IOP ≤ 14 mmHg. Complete success indicated that the target IOP was reached without IOP-lowering medication, whereas it was qualified in cases with or without IOP-lowering medication. Hypotony was defined as IOP < 6 mmHg.

Any additional surgery other than needling procedures was classified as failure, as was loss of light perception. Combinations of IOP-lowering medications were counted as individual treatments.

## Results

Demographic characteristics and clinical findings of the included patients are summarised in Table 1. Four patients had no previous glaucoma surgery. One patient had a single cyclophotocoagulation (CPC) performed two years earlier. One patient had been previously treated with a single selective laser trabeculoplasty (SLT) one year earlier. One patient had received a XEN45 gel stent implantation 2.5 years earlier. One patient was lost to follow-up after one month following surgery. This patient was excluded from further analyses.

**Table 1:** Patients’ Demographics, Baseline Characteristics and Clinical Findings

Case	Age (y)	Glaucoma Type	Previous Surgery	Lens Status	MMC Doses (µg)	Baseline IOP (mmHg)	2-Years IOP (mmHg)	Baseline Medication (n)	2-Years Medication (n)	Needling	TTN (Months)
1	84	POAG	None	Pseudophakic	5	18	n.a.	3	n.a.	No	
2	71	PXG	None	Pseudophakic	5	28	4	4	0	Yes	4
3	54	Pigment	XEN45	Phakic	10	32	13	4	0	No	
4	59	POAG	CPC	Pseudophakic	5	40	15	4	1	No	
5	61	PXG	None	Phakic	5	25	11	3	0	Yes	6
6	66	POAG	None	Phakic	5	40	12	4	0	No	
7	77	POAG	SLT	Pseudophakic	5	39	7	4	0	No	

**Note**: Combinations of IOP-lowering medications were counted as individual treatments.

**Abbreviations**: POAG, primary open-angle glaucoma; PXG, pseudoexfoliative glaucoma; Pigment, pigmentary glaucoma; CPC, cyclophotocoagulation; SLT, selective laser trabeculoplasty; MMC, mitomycin C; TTN, time to first needling.

**Figure 1:**
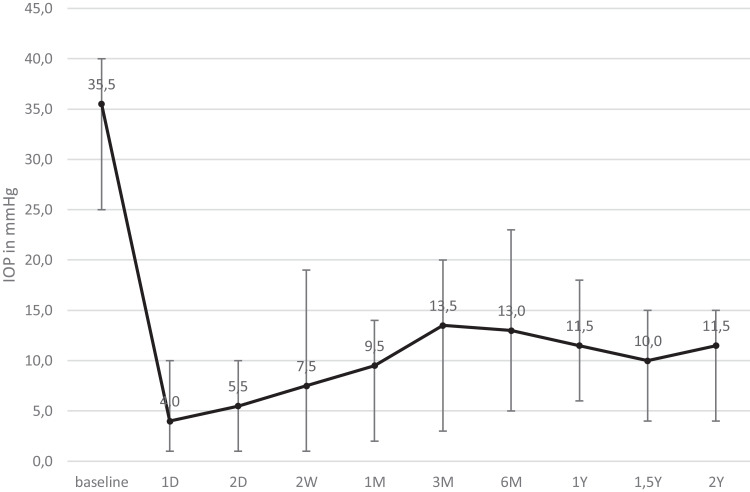
Median intraocular pressure (IOP) at different time points. Whiskers represent range.

Median (range) age was 66 (54.0–84.0) years. Four patients (66.7%) were pseudophakic at the time of surgery. The median IOP decreased from 35.5 mmHg (25.0–40.0 mmHg) from baseline to 11.5 mmHg (4.0–15.0 mmHg) after two years (p = 0.03, Figure 1).

**Figure 2:**
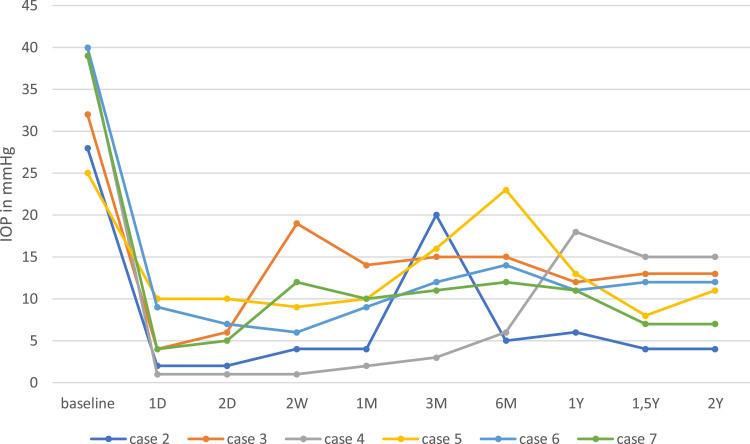
Individual intraocular pressure (IOP) of six of seven patients with a follow-up of two years. In case 2, early postoperative hypotony developed during the first postoperative day. The hypotony persisted over the course of the first week resulting in shallow anterior chamber and choroidal detachment. Healon5 ophthalmic viscoelastic device (OVD) was then injected in the anterior chamber. A second OVD injection was required after two days. The anterior chamber was then reformed and the choroidal detachment resolved completely by the end of the first month even though IOP remained lower than 6.0 mmHg. At month three, IOP increased to 20.0 mmHg due to scarring of the filtering bleb. A single needling procedure with mitomycin C was performed to re-establish outflow. This was followed by a remarkable decrease in IOP, which persisted up to the second year after the XEN63^®^ gel stent implantation. Anterior chamber remained deep; there was no choroidal detachment and the visual acuity recovered fully, so that no further intervention was necessary in this patient. In case 5, a fibrotic bleb developed with an IOP increase to 23.0 mmHg after six months. A single needling procedure was performed which resulted in unmedicated IOP reduction to 13.0 and 11.0 mmHg after one and two years, respectively. In case 7, early postoperative hypotony resulted in shallow anterior chamber and choroidal detachment. Two OVD injections were required during the first two postoperative weeks. Hypotony resolved completely by the end of the second week and no further intervention was necessary up to two years after surgery.

**Figure 3:**
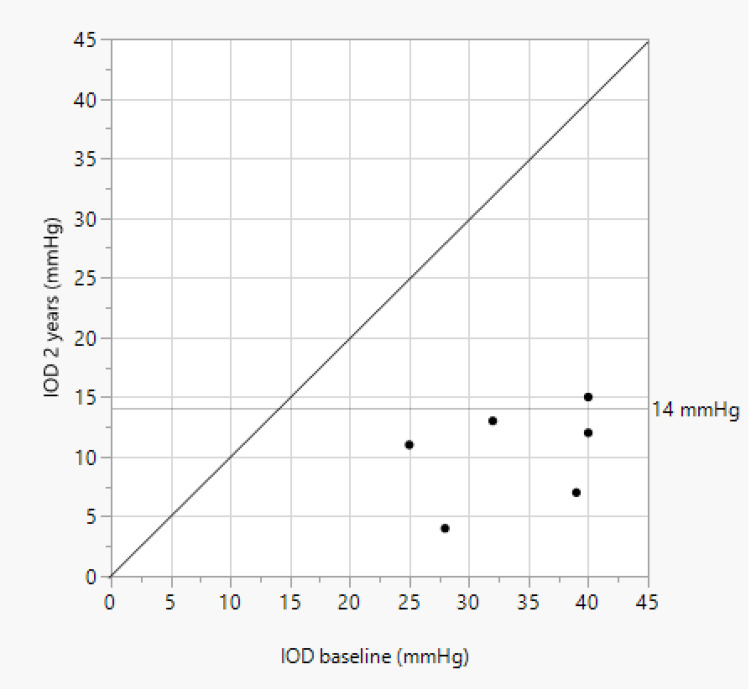
Scatter plot of the baseline and 2-years-postoperative intraocular pressure (IOP).

Median IOP-lowering medication was reduced from 4.0 (3.0–4.0) at baseline to 0 (0–1.0) after two years (p = 0.03). Complete success was reached in five of six patients (83.0%) after 2 years. The one patient who did not have a complete success had an IOP of 15mmHg on a single IOP-lowering medication after two years. Figure 2 shows the individual IOP of the patients at every follow-up time point. Scatter plot of the baseline and 2-years IOP is shown in Figure 3.

**Figure 4:**
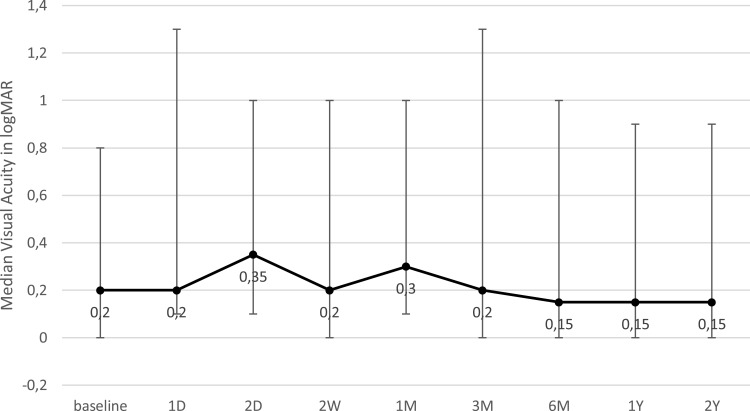
Median visual acuity at different time points. Whiskers represent range.

The median change in visual acuity was −0.05 logMAR (−0.1–0.2) after two years (p = 1.0). None of the patients had a worsening of visual acuity greater than 0.1 logMAR or lost light perception. Figure 4 demonstrates the median visual acuity at follow-up all time points.

Two out of six patients (33.0%) required a single needling procedure because IOP increased over the target after four and six months, respectively.

Four out of six patients (66.7%) had hypotony on the first and the second postoperative day. Two of those had an IOP > 6mmHg at the end of the second week. One patient developed hypotony at day two, which persisted until the end of the first month. Three patients required an intervention due to choroidal detachment and shallowing of the anterior chamber within the first three weeks after surgery. In these patients, the anterior chamber was filled with Healon5 OVD. Two patients required a second injection of Healon5 OVD after two and three days, respectively. Hypotony resolved in all but one patient after three months. In the one patient (16.7%) for whom it did not resolve in that time, the anterior chamber was normal, no choroidal detachment was present and the visual acuity recovered fully without further intervention.

We did not observe any sight-threatening complications.

## Discussion

Our study demonstrated a statistically significant reduction in both IOP and number of IOP-lowering medications after implantation of the new XEN63 in patients with open-angle glaucoma. Complete success was achieved in five of six (83.3%) patients after two years.

Initially, the implantation device of XEN63 gel stent had a 25G needle and required a larger corneal incision compared to the current model.^8^,^9^,^13^ The number of studies on XEN63 is limited and the majority of them have used this early model of the injector, which has never been commercially available.^13–16^ Currently, only one case report and a case series study with a follow-up of 18 months are available which have used the new XEN63 gel stent with a 27G needle.^17^,^18^ To our knowledge, the current study is the first study reporting on the efficacy and safety of the new XEN63 gel stent after two years.

The XEN45 and XEN63 gel stents offer a theoretically derived outflow resistance of 6 to 8 mmHg and 2 to 3 mmHg, respectively, calculated with the Hagen-Poiseuille equation.^8^,^13^ However, actual postoperative IOP also depends on the resistance offered by the subconjunctival space.^19^,^20^ Data from clinical trials confirm this by showing a postoperative IOP level between 12.0 mmHg and 16.8 mmHg for the XEN45.^4^,^21–28^ Data on XEN63 is very limited.^9^,^13–16^,^18^,^29^ Lenzhofer et al reported an IOP reduction from 22.5 ± 4.2 mmHg at baseline to 13.4 ± 3.1 mmHg after four years for the previous version of XEN63 (p < 0.001).^14^ For the same version of the gel stent, Sheybani et al and Lavin-Dapena et al demonstrated a reduction in IOP ≥ 20% without treatment in 47.1% and 54.6% of eyes after a follow-up of one and five years, respectively.^13^,^16^ A complete success (defined as a postoperative IOP ≤ 18.0 mmHg and a reduction in IOP ≥ 20% without medication) was reached in 12 of 53 eyes (25%) and in 3 of 11 eyes (27.3%) in the studies by Lenzhofer et al and Lavin-Dapena et al using the previous version of XEN63.^14^,^16^ Compared to these results, our study demonstrated a median IOP of 11.5 mmHg and a complete success of 83.0% at final follow-up after two years. Significantly higher baseline IOP and a shorter follow-up period in our study are possible explanations for this difference. Another possible explanation is a difference in the study population. In the study by Lenzhofer et al, the population consisted of 87% Caucasians, 6% Asians, 5% Africans and 2% Caribbean compared to only Caucasians in our study. The use of mitomycin to reduce postoperative bleb fibrosis is a major difference between our study and earlier reports on XEN63, in which surgery was performed without antimetabolites. The importance of wound modulation after filtration surgery has been demonstrated repeatedly over the years.^30^ Only one study reported on the new XEN63 with the use of MMC. In it, Fea et al demonstrated a complete success rate comparable to ours (60.9%) which is higher compared to the previous XEN63 without MMC (between 25% and 27.3%).^14^,^16^,^18^

Interestingly, our complete success rate was even higher than that reported by Fea et al, although we used an amount of MMC that was at least four times lower (25µL of MMC 0.02% vs 100µL of MMC 0.02–0.03%).^18^ However, Fea et al included patients with uveitic, traumatic and primary angle-closure glaucoma, which might have negatively influenced their success rate compared to our study.^18^

In our study, two of our six patients (33.0%) required a single needling procedure. In contrast, Fae et al performed needling in four of 23 patients (17.4%).^18^ However, indication for needling might have been different between the studies. In addition, the number of patients included in our pilot trial is low, which limits comparability between the studies.

Interestingly, hypotony on day 1 was more common in our cohort than in the study by Fea et al (66.7% vs 17.4%) even though we used a smaller corneal incision for implantation (0.8 mm vs 1.8 mm) and a smaller amount of MMC (25µL of MMC 0.02% vs 100µL of MMC 0.02–0.03%).^18^ A possible explanation might be a different intensity of removal of the OVD at the end of the procedure. In both studies, hypotony was resolved within up to three months (numerical asymptomatic hypotony persisted in one case in our study), demonstrating that this complication is well manageable. Correspondingly, visual acuity did not change over the course of either study.^18^

Our study has several limitations. The number of patients is low, because of the restricted availability of the new XEN63 at the time this study was performed. Additionally, the oldest patient in our cohort was lost to follow-up one month after surgery, limiting the study population to six patients. Nevertheless, the remaining patients were meticulously followed up on over two years allowing us to gather valuable data on the new XEN63 gel stent.

In conclusion, our study demonstrated a statistically significant reduction in both IOP and number of IOP-lowering medications after implantation of the new XEN63 gel stent in patients with open-angle glaucoma after two years. Complications were well manageable and had no long-term sequelae in this cohort.
